# Hypochlorous Acid-Activated UCNPs-LMB/VQIVYK Multifunctional Nanosystem for Alzheimer’s Disease Treatment

**DOI:** 10.3390/jfb14040207

**Published:** 2023-04-08

**Authors:** Luying Qiao, Yang Shen, Guangzhi Li, Guanglei Lv, Chunxia Li

**Affiliations:** 1 Institute of Molecular Sciences and Engineering, Institute of Frontier and Interdisciplinarity Science, Shandong University, Qingdao 266237, China; 2 Center for Biotechnology and Biomedical Engineering, Yiwu Research Institute of Fudan University, Yiwu 322000, China; 3 College of Pharmacy, Jiamusi University, Jiamusi 154007, China

**Keywords:** upconversion, reactive oxygen species, Alzheimer’s disease, amyloid-β, Tau protein

## Abstract

The development of nanosystems, which can photooxygenate amyloid-β (Aβ), detect the Tau protein, and inhibit effectively the Tau aggregation, is increasingly important in the diagnosis and therapy of Alzheimer’s disease (AD). Herein, UCNPs-LMB/VQIVYK (UCNPs: upconversion nanoparticles, LMB: Leucomethylene blue, and VQIVYK: Biocompatible peptide) is designed as a HOCl-controlled released nanosystem for AD synergistic treatment. Under exposure to high levels of HOCl, the released MB from UCNPs-LMB/VQIVYK will produce singlet oxygen (^1^O_2_) under red light to depolymerize Aβ aggregation and reduce cytotoxicity. Meanwhile, UCNPs-LMB/VQIVYK can act as an inhibitor to decrease Tau-induced neurotoxicity. Besides, UCNPs-LMB/VQIVYK can be used for upconversion luminescence (UCL) due to its unexceptionable luminescence properties. This HOCl-responsive nanosystem offers a new therapy for AD treatment.

## 1. Introduction

Alzheimer’s disease (AD), which gradually influences the physical and mental health of the elderly over 65 years old, is an irreversible and fatal neurodegenerative disease [1,2,3]. Unfortunately, no drugs have been proven to reverse the progress of AD. Hence, most studies focused on slowing down the progression of its deterioration and relieving the pain of the patients. AD has two main hallmarks: the extracellular deposition or misfolding of amyloid-β (Aβ) peptide and the intracellular aggregation of the hyperphosphorylated Tau protein, which lead to the formation of senile plaques and neurofibrillary tangles, respectively [4,5,6]. Aβ42, composed of 42 amino acids, abnormally assembles and rapidly transforms into highly toxic fibrils in AD brains [7,8]. Besides, Tau is a kind of water-soluble protein found primarily in the axons of mature neurons, whereas the hyperphosphorylated Tau protein is water-insoluble and it aggregates in nerve cells, causing attendant cytotoxicity [9,10]. It has been reported that nanoparticles can target Aβ and produce ROS to photooxygenate Aβ [11]. However, the hyperphosphorylated Tau protein also plays a key role in the AD progress. Therefore, nanoparticles that are capable of transporting drugs to the diseased region, suppressing and oxygenating Aβ42 fibers, and targeting and inhibiting hyperphosphorylation Tau at the same time will become a potential therapy for AD treatment. To the best of our knowledge, no nanosystem has been reported to be capable of doing that.

In recent years, more and more nanomaterials have not only been used for in vivo bioimaging [12,13], but have also been used as drug delivery vehicles due to their tiny sizes [14,15], good luminous efficacy, excellent biocompatibility, and high drug-loading efficiency. Among many nanomaterials, the lanthanide-doped upconversion nanoparticles (UCNPs) have become a research hotspot because they have various unique advantages in addition to those mentioned above, such as deep light penetration, multimodal imaging capabilities, superior biostability, and tunable biodistribution [16,17,18,19,20,21,22,23,24,25]. Under near-infrared (NIR) light irradiation, UCNPs can exhibit strong upconversion luminescence (UCL) for locating UCNPs in cells and AD mice [26]. By modifying functionalization ligands, such as poly(etherimide) (PEI), UCNPs can become water-soluble and more biocompatible [27]. Thus, this UCNPs-PEI-based nanovehicle can be combined with UCL imaging for AD therapy.

Several novel phototherapies have emerged for treating human diseases including photodynamic therapy (PDT) and photothermal therapy (PTT) [28,29,30,31]. In addition, photochemical oxygenation and depolymerizing Aβ aggregation by photosensitizing have been reported recently [11,32]. Under red light, methylene blue (MB) will effectively yield singlet oxygen (^1^O_2_) owing to its wonderful photosensitizing property and high singlet oxygen yield [33,34]. Meanwhile, MB can be used as a drug for inhibiting the aggregation of the Tau protein [35,36]. However, because MB has a short systemic half-life and cannot gather in the affected regions, it is vital to devise a medicinal delivery system to transport MB.

**Figure 1:**
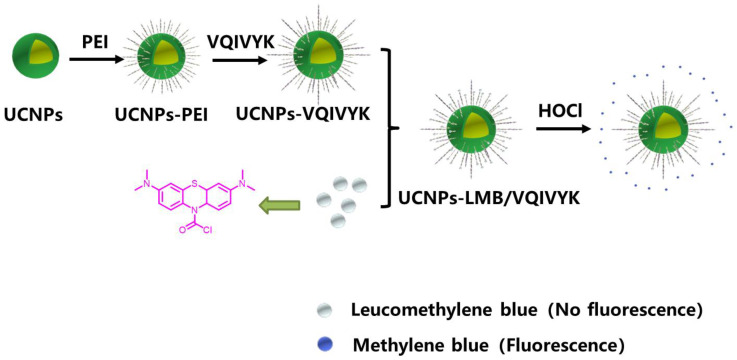
The scheme for the multifunctional treatment platform UCNPs-LMB/VQIVYK.

In this paper, leucomethylene blue (LMB) and biocompatible peptide VQIVYK were conjugated to UCNPs (UCNPs-LMB/VQIVYK) and used as a multifunctional treatment platform for AD treatment (Figure 1). VQIVYK is a key short peptide of the Tau protein, which can be combined with Tau protein with abnormal aggregation trends. Therefore, the coupling of UCNP and VQIVYK can enhance the cell uptake and application effect of UCNP in vivo through receptor-mediated endocytosis [36,37]. MB would be released by HOCl accordingly in the AD brain to inhibit Tau aggregation [38]. Moreover, under red light (>630 nm), the excited MB would produce ^1^O_2_ to suppress and oxygenate Aβ aggregation, resulting in the degradation of Aβ aggregates. More importantly, the released MB can inhibit the aggregation of the Tau protein. Meanwhile, UCNPs with high NIR-to-visible upconversion efficiency can be used for UCL imaging. The Aβ-oxygenating and Tau-inhibiting abilities of UCNPs-LMB/VQIVYK can reduce the cytotoxicity and become a promising treatment for AD.

## 2. Materials and Methods

### 2.1. Synthesis of UCNPs-LMB/VQIVYK

Synthesis of LMB: dichloromethane (DCM, 10 mL) was added to the mixture of methylene blue (MB, 5.00 g, 15.65 mmol, 1.0 eq) and Na_2_CO_3_ (10 g, 94.34 mmol, 6.0 eq), and stirred continuously under the protection of nitrogen at 40 °C. Then, the aqueous solution of sodium hydrosulfite (Na_2_S_2_O_4_, 10.89 g, 12.52 mmol, 0.8 eq) was quickly dropped into the above mixed solution and continuously stirred for 0.5 h. Cooling the reaction solution with an ice water bath until the reactants were layered. Triphosgene (BTC, 3.24 g, 10.88 mmol, 3.47 eq) dissolved in 10 mL DCM was added to the reaction solution and stirred continuously for 2 h in an ice water bath and nitrogen atmosphere. The final reaction solution was extracted with DCM, dried with anhydrous sodium sulfate, evaporated on the rotary evaporator to remove the solvent, and then the product LMB was purified by column chromatography.

LMB (5 mg), EDC (40 mg), and NHS (60 mg) were dissolved in DMF (5 mL) and stirred vigorously for 1 h. UCNPs–PEI (50 mg) was added after the acyl chloride activation. The above solution was mixed for 12 h and UCNPs-LMB was separated by centrifuging.

The peptide VQIVYK was conjugated to UCNPs (UCNPs-LMB/VQIVYK) following a similar method.

### 2.2. ThT Fluorescence Assay

The fibrillation of Aβ42, VQIVYK, and Tau protein was detected by using the fluorescent dye thioflavin T (ThT) with a Hitachi F-4600 Fluorescence Spectrophotometer.

Aβ42 monomer (20 μM) PBS solution was incubated with or without 0.5 mg mL^−1^ UCNPs-LMB/VQIVYK at 37 °C for 48 h. For every 4 h at the first 12 h and every 12 h at the following 36 h, 50 μL of the solution was added into 250 μL of ThT solution (10 μM, in PBS, pH 7.4) and incubated in dark for 30 min. Then, the fluorescence signal (excitation at 440 nm) was recorded at an emission wavelength of 485 nm.

The freeze-dried VQIVYK was dissolved in PBS at a concentration of 1200 μM. The monomerized PBS solution of VQIVYK (1.2 μM, 17 µL) was then added to black 96-well flat-bottomed plates containing ThT (4 mM, 1 µL) in PBS (pH 7.4), sodium heparin (40 μM, 1 µL) in PBS (pH 7.4), UCNPs-LMB/VQIVYK (12 mg mL^−1^, 83 µL) with or without HOCl (4 mM, 2 µL), and PBS to yield final concentrations of 100 µM VQIVYK, 20 µM of ThT, and 0.5 mg mL^−1^ UCNPs-LMB/VQIVYK with or without HOCl (5 μM). The unsealed plates were then placed in a microplate reader (Infinite M200, Tecan, Switzerland) at 37 °C, and the fluorescence of amyloid-bound ThT was monitored with an interval of 1 min for 20 min using excitation and emission wavelengths of 440 and 485 nm, respectively. The plates were shaken for 10 s before each reading with an orbital shaking amplitude of 3 mm.

The freeze-dried Tau was dissolved in PBS (pH 7.4) at a concentration of 4 μM. Briefly, Tau (0.5 μM), ThT (0.5 μM) in PBS (pH 7.4), UCNPs-LMB/VQIVYK + HOCl (0.5 mg mL^−1^), and PBS were added to black 96-well flat-bottomed plates, after which Tau polymerization (aggregation) was initiated by the addition of sodium heparin (3 × 10^−3^ mg mL^−1^) and incubation at 37 °C. The fluorescence of Tau protein-bound ThT was monitored with an interval of 1 h for 24 h using excitation and emission wavelengths of 440 and 485 nm, respectively. The plates were shaken for 10 s before each reading with an orbital shaking amplitude of 3 mm.

### 2.3. Cytotoxicity Assay

To estimate the biocompatibility of UCNPs-LMB/VQIVYK, an MTT cell assay was performed on the PC12 cells.

Firstly, PC12 cells were seeded in 96 well culture plates to grow 24 h in order to divide and expand. Then PC12 cells (5 × 10^3^ per well) were incubated with different concentrations of UCNPs-LMB/VQIVYK for 12 h. After culture, 10 μL MTT solution was added to treat cells as instructed. MTT can bind to succinate dehydrogenase in the mitochondria of living cells to form blue purple crystalline formazan and deposit it in cells, while dead cells do not have this function. After 4 h of treatment, the blue purple crystals were dissolved in dimethyl sulfoxide (DMSO) and their absorption values were measured at a wavelength of 490 nm using a microplate reader [39].

The CCK-8 assay was used to evaluate the cytotoxicity of Aβ42. Firstly, Aβ42 (20 μM) was mixed with UCNPs-LMB/VQIVYK (0.5 mg mL^−1^, with or without HOCl) and pre-treated with 650 nm laser (0.15 W/cm^2^) for 5 min. Then the mixture was co-incubated with PC12 cells for 12 h, whereas only Aβ42 was added as a control group. CCK-8 was added into 96 well plates after 12 h and the absorption values were measured using a microplate reader. The cytotoxicity of Tau was also detected using the CCK-8 assay. Firstly, Tau (0.5 μM) was induced by heparin, and the subsequent processing steps are consistent with those above.

## 3. Results

### 3.1. Synthesis and Characterization of the UCNPs-LMB/VQIVYK Nanosystems

**Figure 2:**
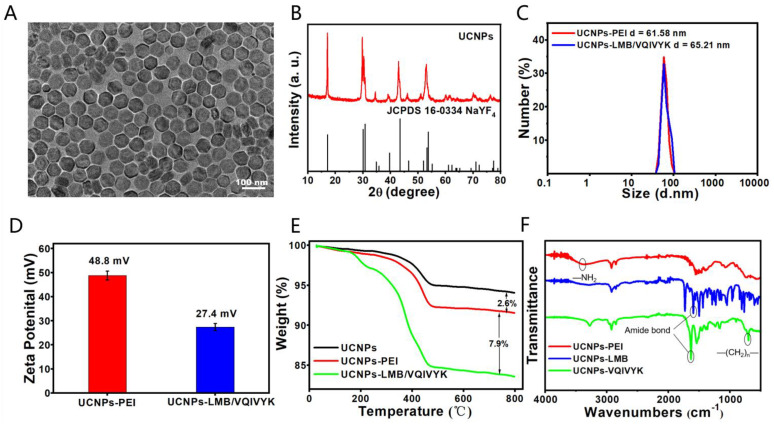
The characterization of UCNPs-LMB/VQIVYK nanosystems: (**A**) TEM image of UCNPs dispersed in cyclohexane, (**B**) XRD patterns of β-NaYF_4_:Yb, Er@NaGdF_4_:Yb nanoparticles (UCNPs). The standard card of β-NaYF_4_ (JCPDS 16-0334) was given as a reference, (**C**) the size distribution and (**D**) zeta potential of UCNPs-PEI and UCNPs-LMB/VQIVYK by dynamic light scattering, (**E**) the thermogravimetric (TGA) result of UCNPs-LMB/VQIVYK, and (**F**) FT-IR spectra of UCNPs-PEI, UCNPs-LMB, and UCNPs-VQIVYK.

The product LMB (white solid, yield 42.5%; ethyl acetate/petroleum ether = 1/10) was purified by column chromatography (Figures S1 and S2 in Supporting Information), ^1^H NMR (400 MHz, CDCl_3_) δ 7.38 (d, J = 8.1 Hz, 2H), 6.69 (d, J = 2.8 Hz, 2H), 6.61 (dd, J = 8.9, 2.8 Hz, 2H), 2.95 (s, 12H). According to the transmission electron microscope (TEM) image and X-ray diffraction (XRD) pattern, as shown in Figure 2A,B, the obtained NaYF_4_:Yb, Er@NaGdF_4_:Yb (UCNPs) had a highly crystalline hexagonal phase with an average diameter of 60 nm. PEI was attached to UCNPs to improve the aqueous solubility [40,41,42,43]. After surface modification, the hydrodynamic size of UCNPs-PEI and UCNPs-LMB was 61.58 and 65.21 nm (UCNPs-LMB), respectively (Figure 2C). The corresponding zeta potentials, which were measured by a Zetasizer Nano-ZS, were 48.8 and 27.4 mV, respectively (Figure 2D). Meanwhile, the thermogravimetric (TGA) results also supported the loading of the PEI coating onto UCNPs decomposed at ≈400 °C upon heating (Figure 2E).

Fourier transform infrared spectrophotometer (FT-IR) spectroscopy also confirmed the existence of amine groups, LMB, and VQIVYK attached to UCNPs (Figure 2F). As shown in the picture, the strong band at 3300 cm^−1^ was assigned to the stretching vibration of NH_2_, suggesting that a large number of NH_2_ groups existed on the surface. The UCNPs-LMB and UCNPs-VQIVYK showed the same bending vibration of the amide bond at 1650 cm^−1^. Besides, the characteristic band of lysine (720 cm^−1^) in VQIVYK appeared, indicating the successful linkage of VQIVYK onto UCNPs.

### 3.2. Drug Loading and Releasing Properties

The oxidation state of MB showed strong absorbance in the red light region(600–700 nm), whereas the reduced state of MB did not have the absorbance in this region. Acyl chloride-modified MB was anchored onto the UCNPs by a facile amide condensation reaction to form UCNPs-LMB/VQIVYK, in which the MB was in a reduced state.

**Figure 3:**
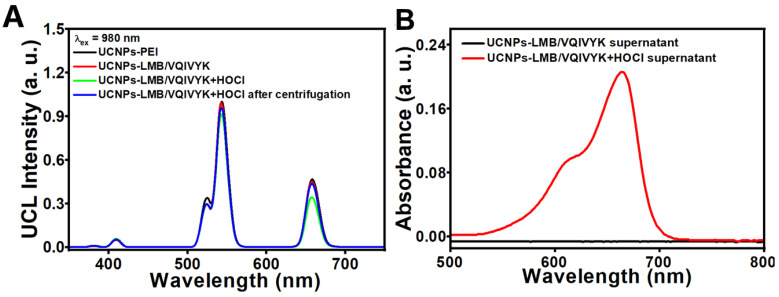
Drug loading and releasing properties: (**A**) the UCL emission spectra of UCNPs-PEI, UCNPs-LMB/VQIVYK, UCNPs-LMB/VQIVYK + HOCl, and UCNPs-LMB/VQIVYK + HOCl dispersed in deionized water after centrifugation at λ_ex_ = 980 nm and (**B**) the UV-vis absorption spectra of the supernatant of UCNPs-LMB/VQIVYK dispersed in deionized water with (red line) or without (black line) HOCl under vigorously stirring for 12 h, UCNPs-LMB/VQIVYK: 0.5 mg mL^−1^.

The observed UCNPs-PEI and UCNPs-LMB/VQIVYK both emitted upconverting visible fluorescence (Figure 3A) of Er^3+^ at 409 nm (^2^H_9/2_→^4^I_15/2_), 521 nm (^2^H_11/2_→^4^I_15/2_), 540 nm (^4^S_3/2_→^4^I_15/2_), and 655 nm (^4^F_9/2_→^4^I_15/2_) under a NIR irradiation of 980 nm. After the addition of HOCl, the peak at 655 nm for UCNPs-LMB/VQIVYK decreased because of the energy transfer between UCNPs and free MB through Förster resonance energy transfer (FRET), and the peak at 655 nm of UCNPs-PEI red emission matched well with the absorbance spectrum of the oxidation state of MB. After the addition of HOCl, the concentration of MB increased to 2.87 μM (Figure 3B), as calculated from the calibration curve (Figure S3 in Supporting Information) of absorbance at 650 nm. Meanwhile, UCNPs-LMB/VQIVYK can be used for UCL imaging, whereas the change of MB absorption can be used for the detection of the existence of HOCl in the AD brain.

### 3.3. Photodynamic Effect of UCNPs-LMB/VQIVYK on Suppressing Aβ42 Aggregation

**Figure 4:**
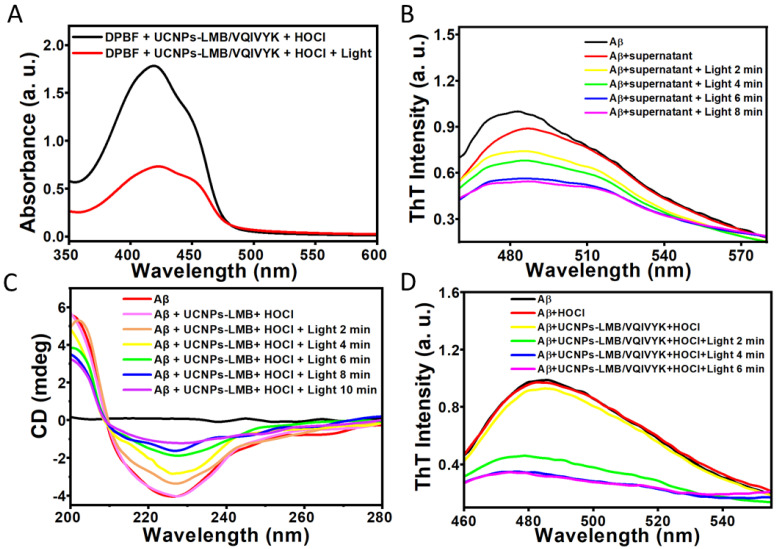
Photodynamic effect of UCNPs-LMB/VQIVYK on suppressing Aβ42 aggregation: (**A**) the variation trend of the absorbance of DPBF mixed with UCNPs-LMB/VQIVYK + HOCl under the 980-nm laser irradiation, (**B**) fluorescence spectra of ThT under different conditions as labeled, (**C**) CD spectra of Aβ under different conditions as labeled, and (**D**) fluorescence spectra of ThT under different conditions as labeled, UCNPs-LMB/VQIVYK: 0.5 mg mL^−1^.

DPBF was selected as a singlet oxygen capture agent and an indicator probe to detect the ^1^O_2_ generation capacity of UCNPs-LMB/VQIVYK nanocomposites (Figure 4A). When UCNPs-LMB/VQIVYK was exposed to the 650-nm light of 0.31 W/cm^2^ in the presence of HOCl, it was found that the absorption intensity of DPBF decreased significantly. This experiment proved that the material has a good ability to generate ^1^O_2_.

As the Aβ42 aggregation could be oxidized with MB under light, we performed multiple photochemical analyses to investigate whether UCNPs-LMB/VQIVYK could inhibit Aβ42 aggregation under NIR. As shown in Figure 4B,D, the thioflavin T (ThT, for detecting Aβ42 and Tau aggregation [44,45]) showed that the dissociated MB strongly suppressed Aβ42 aggregation after the addition of HOCl upon 4 min of NIR irradiation. However, according to the control experiment (Figure S4 in Supporting Information), NIR has no effect on Aβ42 protein aggregation. Then, the UCNPs-LMB/VQIVYK and HOCl mixture was centrifuged to obtain the supernatant containing MB. The ThT assay and circular dichroism (CD) (Figure 4C) showed that the photosensitized MB photooxygenated Aβ42 aggregates. When Aβ42 fibrils were incubated with UCNPs-LMB/VQIVYK + HOCl, the CD spectrum exhibited large positive and negative peaks at 200 and 226 nm, respectively, which can be attributed to the typical β-sheet secondary structure. Under NIR illumination, the negative peak gradually disappeared in a time-dependent manner. These results corroborated that Aβ42 aggregates can be disintegrated by NIR-induced UCNPs-LMB/VQIVYK.

### 3.4. UCNPs-LMB/VQIVYK Restrained Tau Protein Aggregation

In order to observe the suppression effect of MB on Tau aggregation, we first performed multiple analyses on a six amino acid peptide VQIVYK. Meanwhile, the aggregation of VQIVYK fragments is similar to those generated by the full-length Tau [46,47]. Therefore, we chose this fragment as a simple model system [35,37,48,49,50,51,52].

**Figure 5:**
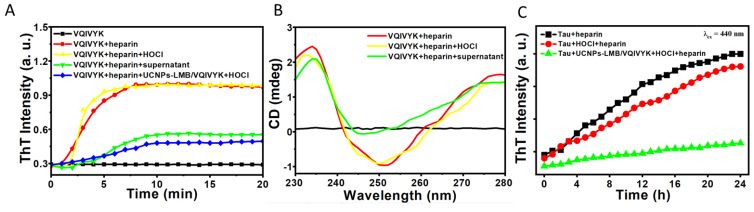
The effects of UCNPs-LMB/VQIVYK on Tau protein aggregation: (**A**) fluorescence spectra of ThT, (**B**) CD spectra of VQIVYK under different conditions, and (**C**) the kinetics of heparin-induced Tau protein formation without or with the addition of UCNPs-LMB/VQIVYK, and HOCl were monitored by ThT fluorescence from 0 to 24 h, with Tau: 0.5 μM, heparin: 3 × 10^−3^ mg mL^−1^, UCNPs-LMB/VQIVYK: 0.5 mg mL^−1^, HOCl: 10 μM, λ_ex_ = 440 nm, and λ_em_ = 495 nm.

First, we performed kinetic ThT experiments to demonstrate that UCNPs-LMB/VQIVYK can inhibit VQIVYK aggregation in the absence of heparin (Figure 5A). The fluorescence of VQIVYK in the absence of heparin increased rapidly within 8 min and reached a plateau, whereas both UCNPs-LMB/VQIVYK + HOCl and supernate with MB can inhibit the rate of VQIVYK aggregation by reducing the slope to decrease ThT fluorescence. Besides, HOCl was proved to have no influence on the VQIVYK aggregation. Next, we used CD spectroscopy to investigate the secondary structure of VQIVYK in different conditions. Figure 5B shows that in the presence of heparin VQIVYK adopted a β-sheet structure characterized by a negative peak at 250 nm. After the addition of supernatant, the CD results showed a reduced positive peak, indicating the ability of UCNPs-LMB/VQIVYK to suppress VQIVYK aggregation.

Then, the effect of UCNPs-LMB/VQIVYK on inhibiting Tau aggregation was studied by ThT assay. Heparin-induced Tau aggregation increased rapidly within 20 h (Figure 5C). The dissociative MB from the mixture of UCNPs-LMB/VQIVYK can reduce the level of ThT fluorescence caused by Tau protein aggregation. These results further demonstrated the efficient anti-aggregation ability of UCNPs-LMB/VQIVYK.

### 3.5. Release of MB in Cells

In order to evaluate whether UCNPs-LMB/VQIVYK nanocomposites can release MB in response to endogenous HOCl in PC12 cells, the fluorescence intensity of MB in cells was first detected by an enzyme microplate meter. As illustrated in Figure S5 (Supporting Information), when the PC12 cells were co-incubated with UCNPs-LMB/VQIVYK at different time, only a small amount of released MB could be detected and the fluorescence intensity of MB increased slightly with time. When pre-incubated with LPS for 15 min, the PC12 cells were induced to produce endogenous HOCl. It was found that the fluorescence intensity of MB increased significantly with the extension of time. This experiment confirmed that UCNPs-LMB/VQIVYK nanocomposites can react with endogenous HOCl to release MB.

**Figure 6:**
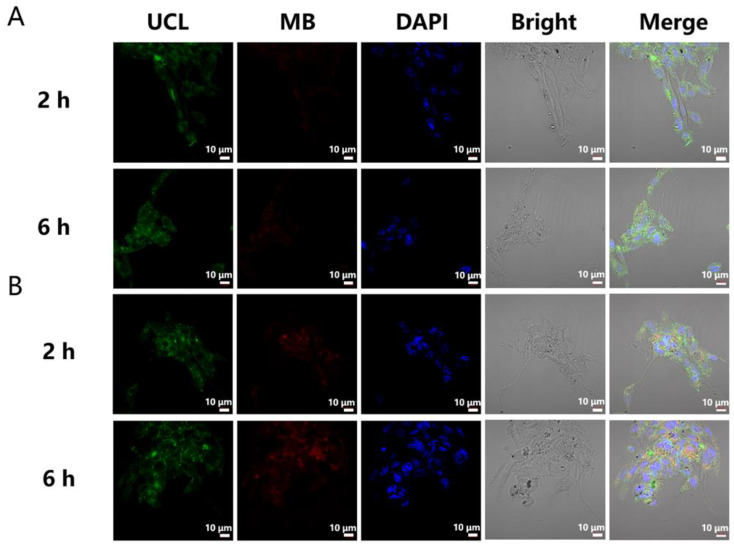
The effects of LPS on the MB release of UCNPs-LMB/VQIVYK in PC12 cells. PC12 cells were incubated (**A**) without and (**B**) with LPS for 15 min, and then incubated with UCNPs-LMB/VQIVYK for 2 and 6 h for confocal fluorescence imaging, with UCL (λ_ex_ = 980 nm, 540 ± 50 nm), MB (λ_ex_ = 650 nm, 685 ± 50 nm), DAPI, merged images, UCNPs-LMB/VQIVYK: 0.5 mg mL^−1^, and scale bar: 10 μm.

In order to further evaluate the ability of UCNPs-LMB/VQIVYK nanocomposites to release MB in cells, confocal laser scanning microscopy was selected to image PC12 cells. When the PC12 cells were incubated with UCNPs-LMB/VQIVYK for 1 h, the fresh medium was replaced. After 2 and 6 h of incubation, fluorescent cell images were obtained (Figure 6A). With the prolongation of incubation time, no obvious MB fluorescence signal was observed whereas an obvious UCNPs fluorescence signal was observed, which proved that UCNPs-LMB/VQIVYK nanocomposites had good cell membrane penetration and cell imaging ability, and they could enter PC12 cells although no MB was released. When the PC12 cells were incubated with UCNPs-LMB/VQIVYK for 1 h, the fresh medium was replaced, and LPS and PC12 cells were incubated for 15 min. Then, they were incubated for 2 and 6 h, and the fluorescent cell images were obtained (Figure 6B). With the prolongation of incubation time, obvious UCNPs fluorescence signal and MB fluorescence signal can be observed and they increased gradually, which evidenced that the PC12 cells produce endogenous HOCl in the presence of LPS, and UCNPs-LMB/VQIVYK nanocomposites can release MB.

### 3.6. UCNPs-LMB/VQIVYK Reduced the Cytotoxicity of Aβ42/Tau

**Figure 7:**
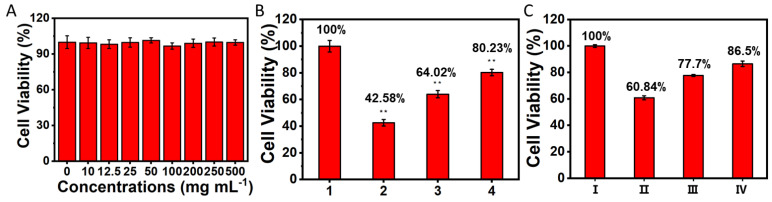
The effects of UCNPs-LMB/VQIVYK on the cytotoxicity of Aβ42 aggregates/Tau protein: (**A**) the survival rate of PC12 cells incubated with UCNPs-LMB/VQIVYK nanocomposites (0 to 500 μg/mL) for 12 h, as measured by the MTT method. The CCK8 experiment on the survival rate of PC12 cells incubated with UCNPs-LMB/VQIVYK and (**B**) Aβ42 aggregates or (**C**) Tau protein with or without HOCl. 1: control group; 2: Aβ42; 3: Aβ42 + UCNPs-LMB/VQIVYK; 4: Aβ42 + UCNPs-LMB/VQIVYK + HOCl; Ⅰ: control group; Ⅱ: Tau; Ⅲ: Tau + UCNPs-LMB/VQIVYK; and Ⅳ: Tau + UCNPs-LMB/VQIVYK + HOCl, UCNPs-LMB/VQIVYK: 0.5 mg mL^−1^. Statistical significance is assessed by an unpaired Student’s two-sided t-test. ** *p* < 0.01.

In order to evaluate the biological safety of UCNPs-LMB/VQIVYK nanocomposites, we first tested the effect of different concentrations of UCNPs-LMB/VQIVYK on the survival rate of PC12 cells by the MTT method. As shown in Figure 7A, the PC12 cells and UCNPs-LMB/VQIVYK nanocomposites (0 to 500 μg/mL) were cultivated together for 12 h, and the cell survival rate was above 90%. It proved that the toxicity of UCNPs-LMB/VQIVYK nanocomposites was very low, and they are suitable for biological experiments. Next, we studied whether UCNPs-LMB/VQIVYK nanocomposites could reduce the cytotoxicity of Aβ42 aggregates under different conditions by measuring the cell survival rate. The CCK8 experiment (Figure 7B) was used to investigate the cell survival rate of PC12 cells under different conditions. The control group was the cells incubated with a normal culture medium, and their cell activity was 100%. When the PC12 cells were cultivated with Aβ42 for 12 h, the survival rate of PC12 cells decreased to 42.58%. When the Aβ42 aggregates were mixed with UCNPs-LMB/VQIVYK (with or without HOCl) and pretreated with a 650 nm laser (0.15 W/cm^2^) for 5 min. The mixture was incubated with PC12 cells for 12 h, and the cell survival rate increased to 80.23% and 64.02%, respectively. This implied that in the presence of Aβ42-aggregates-induced cytotoxicity, UCNPs-LMB/VQIVYK also had a certain protective effect on PC12 cells. However, in contrast, in the presence of HOCl, it can induce UCNPs-LMB/VQIVYK to release MB in large quantities, thus degrading Aβ42 aggregates and effectively protecting PC12 cells from the toxic effects of Aβ42 aggregates. In addition, the effects of UCNPs-LMB/VQIVYK nanocomposites on reducing the cytotoxicity of Tau aggregates were studied. As shown in Figure 7C, when the heparin-induced Tau protein aggregates and PC12 cells were incubated together for 12 h, the cell survival rate decreased to 60.84% relative to the control group. When the heparin-induced Tau protein was mixed with UCNPs-LMB/VQIVYK (with or without HOCl) and incubated with PC12 cells for 12 h, the cell survival rate increased to 86.50% and 77.70%, respectively. This indicated that UCNPs-LMB/VQIVYK also had a certain protective effect on PC12 cells in the face of cytotoxicity induced by Tau aggregates. But in contrast, in the presence of HOCl, it can induce UCNPs-LMB/VQIVYK to release MB in large quantities, enhance the effect of inhibiting Tau protein aggregation, and effectively protect PC12 cells from the toxic effect of Tau aggregates.

## 4. Discussion

At present, the pathogenesis of Alzheimer’s disease is still unclear, which makes it hard to find an effective cure for this disease. Therefore, the realization of effective diagnosis and treatment for Alzheimer’s disease is a significant challenge for the scientific community [53,54]. In recent years, with the development of nanotechnology and the continuous intersection of nanotechnology and biomedicine, more and more researchers have turned their attention to the application of nanomaterials in the diagnosis and treatment of Alzheimer’s disease [55]. Among many nanomaterials, lanthanide-doped upconversion nanoparticles (NaYF_4_: 20% Yb and 2% Er@NaGdF_4_: 2% Yb) have been selected as luminescent nuclei and drug carriers due to their good luminescent stability, deep penetration, and multimodal imaging capabilities [39]. Because UCNPs have poor water solubility and need to be modified with functional ligands on their surfaces to improve their water solubility and biocompatibility. We chose polyethylene imine (PEI) to replace the OA portion of the surface of UCNPs. UCNPs-LMB/VQIVYK was synthesized by covalently linking the reduced MB to the surface of UCNPs-NH_2_ using coupling reagents EDC and NHS. The UCNPs-LMB multifunctional nanocomposite can be excited by the 980 nm NIR light. Because of the overexpressed reactive oxygen species in the AD patients’ brains, this multifunctional nanocomposite can respond to HOCl rapidly, leading to the controlled release of fluorophore MB, and it acted as an effective inhibitor for inhibiting Tau protein aggregation, as proved by the ThT fluorescence test and circular dichroism test [56]. Er^3+^ was doped into UCNPs, and it can be used as an activator for converting the 980 nm NIR light to visible light at 655 nm. The 655 nm light activated photosensitizer MB for producing singlet oxygen (^1^O_2_), and it can degrade Aβ aggregates and reduce its biotoxicity to the surrounding tissues. Both in vitro and cell experiments showed that the UCNPs-LMB multifunctional nanocomposite could respond to HOCl rapidly and release MB for effectively inhibiting Tau protein aggregation and photo-depolymerizing Aβ aggregates under NIR light irradiation. The cytotoxicity assay showed that the UCNPs-LMB multifunctional nanocomposite had high biocompatibility. More importantly, UCNPs-LMB can reduce the cytotoxicity of AD pathological protein to PC12 cells and improve cell viability. A novel HOCl-responded drug delivery nanosystem (UCNPs-LMB/VQIVYK) was successfully designed, and it will provide a new diagnosis and treatment system for AD treatment.

## 5. Conclusions

A nanosystem UCNPs-LMB/VQIVYK has been successfully developed, and it is capable of oxygenating Aβ42 fibers and inhibiting hyperphosphorylation Tau at the same time. A series of characterizations have been carried out to verify the successful synthesis of the material and the potential ability to treat AD through ThT fluorescence experiment and CD spectrum. Our work can provide a new viewpoint on AD therapy.

## Data Availability

The corresponding author will make the data available upon reasonable request.

## Supplementary Materials

The following supporting information can be downloaded at: https://www.mdpi.com/article/10.3390/jfb14040207/s1, Figure S1. Synthesis roadmap of LMB; Figure S2. ^1^H NMR of LMB in CDCl_3_; Figure S3. The standard curve of MB in aqueous solution; Figure S4. Effect of NIR on ThT fluorescence of Aβ42 protein aggregation; Figure S5. MB release test of UCNPs-LMB in PC12 cells with or without LPS. Table S1. English acronym meaning and full name.
