# Supplementary material for: Hypochlorous Acid-Activated UCNPs-LMB/VQIVYK Multifunctional Nanosystem for Alzheimer’s Disease Treatment
Source: J Funct Biomater. 2023 Apr 8;14(4):207. doi: 10.3390/jfb14040207 (PMC10143957; doi:10.3390/jfb14040207)
Supplement: Supplementary file 1 [file jfb-14-00207-s001.zip › jfb-2199295-supplementary.pdf]

# Hypochlorous acid-activated UCNPs-LMB/VQIVYK Multifunctional Nanosystem for Alzheimer's Disease Treatment

**Luying Qiao[1], Yang Shen[2], Guangzhi Li[3], Guanglei Lv[2,*] and Chunxia Li[1,*]**

[1] Institute of Molecular Sciences and Engineering, Institute of Frontier and Interdisciplinarity Science, Shandong University, Qingdao 266237, P. R. China; cxli@sdu.edu.cn
[2] Center for Biotechnology and Biomedical Engineering,Yiwu Research Institute of Fudan University,Yiwu 322000, P. R. China; lvguanglei@fudan.edu.cn
[3] College of Pharmacy, Jiamusi University, Jiamusi 154007, P. R. China

* Correspondence: cxli@sdu.edu.cn, lvguanglei@fudan.edu.cn

## EXPERIMENTAL SECTION

## Materials

All the chemical reagents are used as received without further purification. Rare earth oxides ($Y_2O_3$, $Gd_2O_3$, $Yb_2O_3$, and $Er_2O_3$) and rare earth chlorides ($YCl_3$, $YbCl_3$, and $ErCl_3$) were purchased from Beijing HWRK Chem Co., LTD. The $Gd(CF_3COO)_3$ and $Yb(CF_3COO)_3$ solutions were prepared by dissolving the corresponding rare earth oxides in a trifluoroacetic acid aqueous solution, followed by removal of the trifluoroacetic acid through evaporation. Oleic acid (OA, 90%, technical grade), polyetherimide (PEI), N-hydroxysuccinimide (NHS, 98%), N-(3-dimethylaminopropyl)-N-ethylcarbodiimide hydrochloride (EDC, 98%), thiazolyl blue tetrazolium bromide (MTT) were purchased from Sigma-Aldrich. Trifluoroacetic acid ($CF_3COOH$) and octadecene (ODE, 90%, technical grade) were purchased from J&K. Ethanol (≥99.7%), hypochlorous acid (≥30%) and dimethyl sulfoxide（DMSO）were purchased from Sinopharm Chemical Reagent Co., Ltd. Ammonium nitrate ($NH_4NO_3$, ≥99.0%), $CF_3COONa$, hexafluoroisopropanol (HFIP), NaOH (reagent grade, ≥98%), ammonium fluoride ($NH_4F$), methylene blue (MB), heparin sodium, and thioflavine T (ThT) were purchased from Aladdin. β-amyloid peptides (Aβ42) and VQIVYK were provided by GL Biochem Ltd. (Shanghai, China). Recombinant human Tau protein were purchased from R&D. DMEM, Fetal bovine serum (FBS) and horse serum were purchased from Gibco (Life Technologies AG, Switzerland). PC12 cells (rat pheochromocytoma, American Type Culture Collection), purchased from HangZhou Hibio Technology CO.,LTD, were cultured in DMEM medium supplemented with 10% FBS at 37°C under a humidified atmosphere containing 5% $CO_2$.

## Synthesis of UCNPs-PEI

$NaYF_4$:Yb, Er@$NaGdF_4$:Yb core/shell UCNPs were prepared following the literature method with slight modification[1]. First, hexagonal phase (β-) $NaYF_4$:Yb, Er (78% Y, 20% Yb, and 2% Er) cores were synthesized via a thermal decomposition reaction.

$YCl_3$ (780 μmol), $YbCl_3$ (200 μmol), $ErCl_3$ (20 μmol) were dissolved in 20 mL of OA/ODE (v/v=1:1) and heated to 140 °C and then cooled to 40 °C. Then, NaOH (100 mg) and $NH_4F$ (150 mg) dissolved in 10 mL of methanol were added into the mixture above. Next, the mixture was vacuumized for 30 min at 110 °C and reacted at 300 °C under a $N_2$ atmosphere for 1 h. The obtained parent cores were dissolved in 10 mL cyclohexane solution.

Under vigorously stirring, 5 mL of cyclohexane solution containing half of the cores was added into 20 mL of OA/ODE (v/v=1:1). The mixture was heated to 150 °C under vacuum, and the rapidly heated to 310 °C. 1 mmol of $RE(CF_3COO)_3$ (98% $Gd^{3+}$ and 2% $Yb^{3+}$, molar ratio) and 1mmol of $CF_3COONa$ mixed with 4 mL of OA/ODE (v/v= 1:1) as shell precursors were injected into the above solution immediately.

UCNPs-PEI nanoparticles were synthesized according to the previous procedures[2]. 0.5 g of PEI and 10 mL of distilled water were added into 2 mL of trichloromethane solution containing 100 mg of OA-UCNPs. The mixture was stirred vigorously at room temperature for 24 h to evaporate the trichloromethane, producing a clear and transparent UCNPs–PEI

water solution. Then the mixture was centrifuged and washed three times with distilled water.

## Characterization of UCNPs-PEI and UCNPs-LMB/VQIVYK

The obtained products were characterized by using various methods. The morphology of the UCNPs was determined using transmission electron microscope (TEM, Hitachi, H-7650). TEM samples were prepared by dispersing the nanoparticles solution onto a holey carbon film on copper grids. Zeta-potential and size distribution of UCNPs-PEI and UCNPs-LMB were determined on a Nano-ZS instrument (Malvern Instruments Limited). Optical absorption spectra were measured with a UV–vis spectrophotometer (UH5300 UV-Vis). All emission spectra were recorded by a Hitachi F-4600 Fluorescence Spectrophotometer with a scan speed of 240 nm/min. Powder X-ray diffraction (PXRD) patterns of the UCNPs-PEI, UCNPs-LMB and UCNPs-VQIVYK powder were taken using a MAXima_X XRD-7000 diffractometer equipped with Cu K$\alpha$ ($\lambda$ = 1.542 Å) radiation under room conditions. The Fourier transform infrared spectrum (FT-IR) of the sample was recorded on the Equinox 55 infrared spectrometer by using the compression method.

## Drug Loading and Releasing Properties of UCNPs-LMB/VQIVYK

In vitro drug storage of UCNPs-LMB/VQIVYK was carried out by using EDC-NHS reaction under magnetic stirring for 24 h at 37 °C. The obtained UCNPs-LMB/VQIVYK was collected by centrifugation and dispersed in 10 mL in pH = 7.0 phosphoric acidic buffer solutions (PBS). After that the UCNPs-LMB/VQIVYK solution was added with 10 μM HOCl under magnetic stirring at 37 °C, respectively. After 1 h, aliquots were taken from the suspension and centrifuged. The supernatants were measured by UV–vis spectrum and fluorescence spectrum. The absorbance band and emission band of the MB centered at 650 nm and 695 nm.

## Preparation of Aβ42

Aβ42 was prepared following a previous protocol[3]. At first, Aβ42 monomer was dissolved in HFIP and stored at -20 °C as the stock solution. Before use, the Aβ42 solution was freeze-dried under high vacuum and dissolved in PBS (pH 7.4) to a concentration of 100 μM.

## Cell culture

PC12 cells (rat pheochromocytoma) were cultured in Iscove modified Dulbecco me-dium (IMDM) supplemented with 5% foetal bovine serum, 10% horse serum at 37 °C in the $CO_2$ (5%) environment.

## Upconversion luminescence imaging of UCNPs-LMB/VQIVYK and MB release

MB release was detected in PC12 cells. 1 μg $mL^{-1}$ lipopolysaccharide (LPS) was coincubated with PC12 cells for 15 min, and then coincubated with 0.5 mg $mL^{-1}$ UCNPs-LMB/VQIVYK for 2 and 6 h. Without LPS and only adding UCNPs-LMB/VQIVYK as a control. Then confocal laser scanning microscopy (CLSM) was used to detect MB fluorescence intensity.

# RESULTS

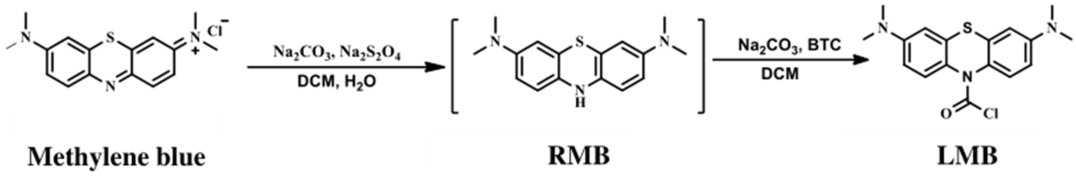


Figure S1. Synthesis roadmap of LMB.

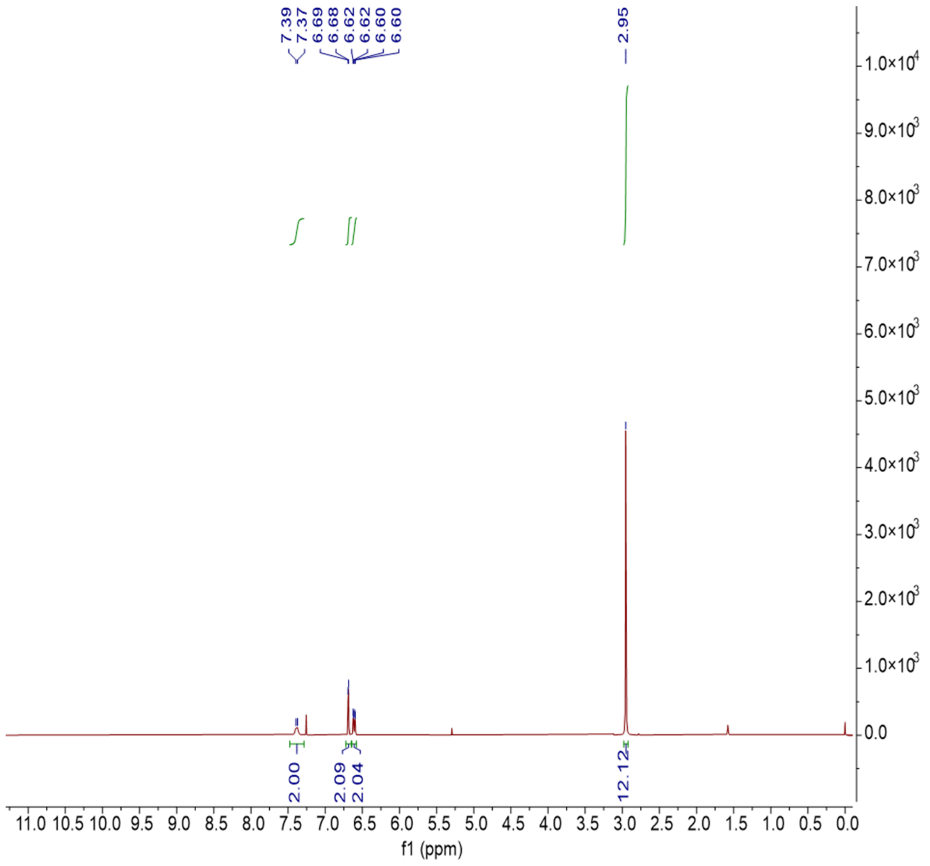


Figure S2. $^1$H NMR of LMB in $CDCl_3$.

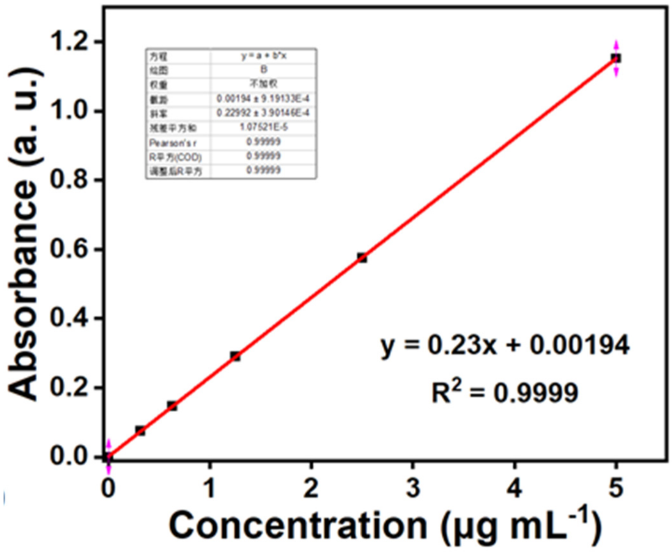


Figure S3. The standard curve of MB in aqueous solution.

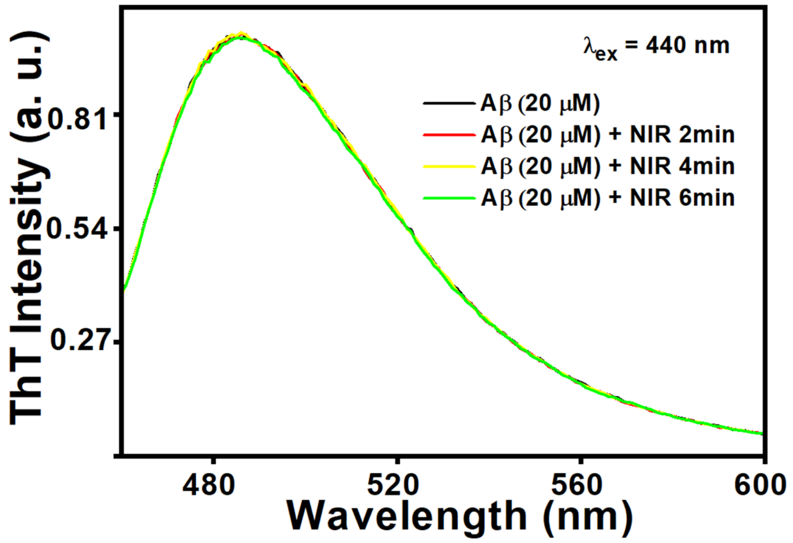


Figure S4. Effect of NIR on ThT fluorescence of Aβ42 protein aggregation.

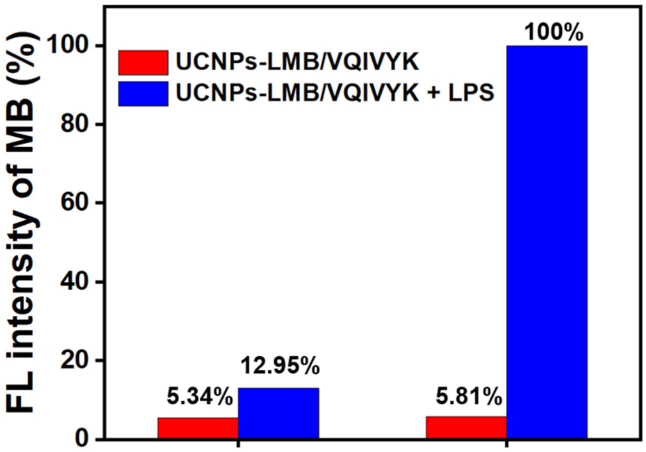


Figure S5. MB release test of UCNPs-LMB/VQIVYK in PC12 cells with or without LPS.

| **Acronym Meaning** | **Full name** |
|---|---|
| AD | Alzheimer's disease |
| UCNPs | upconversion nanoparticles |
| LMB | Leucomethylene blue |
| VQIVYK | Biocompatible peptide |
| $^1O_2$ | singlet oxygen |
| UCL | upconversion luminescence |
| Aβ | amyloid-β peptide |
| NIR | near-infrared |
| PEI | poly(etherimide) |
| PDT: | photodynamic therapy |
| PTT | photothermal therapy |
| MB | methylene blue |
| DCM | dichloromethane |
| TEM | transmission electron microscope |
| XRD | X-ray diffraction |
| TGA | thermogravimetric |
| FT-IR | Fourier transform infrared spectrophotometer |
| FRET | Förster resonance energy transfer |

| | |
|---|---|
| ThT | thioflavin T |
| CD | circular dichroism |
| EDC | N-(3-dimethylaminopropyl)-N-ethylcarbodiimide hydrochloride |
| NHS | N-hydroxysuccinimide |
| HFIP | hexafluoroisopropanol |
| DMSO | dimethyl sulfoxide |
| MTT | tetrazolium bromide |
| OA | Oleic acid |
| ODE | octadecene |
| FBS | Fetal bovine serum |
| PBS | phosphoric acidic buffer solutions |
| CLSM | confocal laser scanning microscopy |
| LPS | lipopolysaccharide |

Table S1. English acronym meaning and full name.

## References

1. Wang, M.; Chang, M.Y.; Li, C.X., Chen, Q.; Hou, Z.Y.; Xing, B.G.; Lin, J. Tumor-Microenvironment-Activated Reactive Oxygen Species Amplifier for Enzymatic Cascade Cancer Starvation/Chemodynamic/Immunotherapy. *Adv Mater* **2022**, 34, 2106010, doi:10.1002/adma.202106010.
2. Hou, Z.; Zhang, Y.; Deng, K.; Chen, Y.; Li, X.; Deng, X.; Cheng, Z.; Lian, H.; Li, C.; Lin, J. UV-Emitting Upconversion-Based $TiO_2$ Photosensitizing Nanoplatform: Near-Infrared Light Mediated in Vivo Photodynamic Therapy via Mitochondria-Involved Apoptosis Pathway. *ACS nano* **2015**, 9, 2584-2599, doi:10.1021/nn506107c.
3. Teng, B.; Han, Y.; Zhang, X.; Xiao, H.; Yu, C.; Li, H.; Cheng, Z.; Jin, D.; Wong, K.L.; Ma, P.; et al. Phenanthriplatin(iv) conjugated multifunctional up-converting nanoparticles for drug delivery and biomedical imaging. *J Mater Chem B* **2018**, *6*, 5059-5068, doi:10.1039/c8tb01034j.
